# Cardio Cerebral Resuscitation: Is it better than CPR?

**Published:** 2009-12

**Authors:** TVSP Murthy, Bhavna Hooda

**Affiliations:** 1 Prof & Senior Adviser, Dept of Anaesthesia and Critical Care, Command Hospital (CC), Lucknow - 226002; 2 Graded Specialist, Dept of Anaesthesia and Critical Care, Command Hospital (CC), Lucknow - 226002

**Keywords:** Cardio cerebral Resuscitation, CPR, Prehospital cardiac arrest

## Abstract

The guidelines for cardiopulmonary resuscitation (CPR) have been in place for decades; but despite their international scope and periodic updates, there has been little improvement in survival rates in out-of-hospital cardiac arrest for patients who did not receive early defibrillation. Instituting the new cardio cerebral resuscitation protocol for managing prehospital cardiac arrest improved survival of adult patients with witnessed cardiac arrest and an initially shockable rhythm.

## Introduction

Cardiac arrest highlights one of the critical interactions between the heart and the brain, and it remains a leading cause of death. The concept of cardio cerebral resuscitation as an alternative to traditional cardiopulmonary respiration (CPR) for out-of-hospital cardiac arrest is fast evolving into a reality. Because cardio cerebral resuscitation results in improved survival and cerebral function in patients with witnessed cardiac arrest with a shockable rhythm, it should replace CPR for out-of-hospital cardiac arrest and CPR should be reserved for respiratory arrest.^1^

## The need for replacement: CCR in place of CPR

Despite the development and periodic updating of guidelines for CPR and emergency cardiovascular care from the American Heart Association (AHA) survival rates for victims of out-of-hospital cardiac arrest are dismal and have remained essentially unchanged in the recent past.^2^,^3^

**The traditional CPR** approach has three major drawbacks:

- Most bystanders to a person who unexpectedly collapses are willing to activate emergency medical services (EMS) but are not willing to initiate rescue efforts because they do not want to perform mouth-to mouth assisted ventilation. Bystanders are more willing to perform chest-compression-only resuscitation for a person who unexpectedly collapses an approach that all agree is dramatically better than doing nothing.
- Interrupting chest compressions for ventilation during cardiac arrest decreases survival.
- Positive pressure ventilation during CPR for cardiac arrest increases intrathoracic pressures, which decreases venous return to the thorax and subsequent perfusion of the heart and the brain.^4^,^5^

## Cardiocerebral Resuscitation Eliminates Ventilation

In contrast to CPR, cardio cerebral resuscitation eliminates mouth-to-mouth ventilation for bystanderinitiated resuscitation efforts, dramatically decreases the role of positive pressure ventilation by EMS responders, and emphasizes chest compressions prior to and immediately after a single shock for cardiac arrests not witnessed by EMS personnel.

## The evidence base

In a human study, investigators from Japan found that among witnessed victims of out-of-hospital cardiac arrest who had a shockable rhythm upon the arrival of EMS personnel, chest-compression-only resuscitation resulted in better survival than did chest compressions plus mouth-to-mouth ventilation.^6^

## What the public should be taught about resuscitation

The message that needs to be promulgated is twofold but nevertheless simple: firstly-cardio cerebral resuscitation is for cardiac arrest, and secondly CPR with ventilation is recommended for respiratory arrest. The lay public should be taught that an unexpected collapse in an adult is, in all likelihood, a cardiac arrest, to be differentiated from obvious respiratory arrest, such as choking or drowning, where assisted ventilations may be appropriate.^4^

## Coronary Perfusion Pressure Is Essential During Prolonged Cardiac Arrest

In the absence of early defibrillation, survival beyond the first 5 minutes of ventricular fibrillation (VF) arrest is predominantly dependent on adequate coronary and cerebral perfusion pressures, both of which are generated by chest compressions. It is well established that in the absence of early defibrillation or bystander-initiated resuscitation efforts, survival is rare.

The decades-old recommendation of two ventilations before each 15 chest compressions has recently been acknowledged not to be optimal, as this ratio was changed from 2:15 to 2:30 in the 2005 AHA guidelines to increase the recommended number of chest compressions. However, this change did not address the major problem, which is bystanders’ reluctance to initiate resuscitation if ventilation is involved, regardless of the ventilations-to-compressions ratio. The greatest impediment to the initiation of bystander resuscitation is the public's aversion to and/or the complicated nature of performing mouth-to-mouth resuscitation.^5^,^6^

## The Role of Gasping or Agonal Respirations:

When a person collapses with VF, or if VF is induced in an animal model, gasping is present in a significant number of individuals and animals. This abnormal breathing, which varies in duration, can be either fortunate or unfortunate. When chest compressions are promptly initiated, gasping is fortunate in that the subject is likely to continue to gasp and provide self ventilation (negative intrathoracic pressure)^7^.

However, gasping also may be unfortunate in that most laypersons interpret it as an indication that the subject is still breathing, causing them not to initiate bystander resuscitation or call for EMS personnel as soon as they should. Education will be essential to ensure prompt initiation of bystander chest compressions in patients who gasp with cardiac arrest, as well as to ensure that chest compressions are not stopped because of continued gasping.

## Implementing Cardiocerebral Resuscitation Into EMS Protocols

In emergency medical service protocols, laypersons are to be taught to “be a lifesaver.” They are to be instructed to call emergency as soon as possible and then to begin chest compressions alone. If an automated external defibrillator (AED) is available, they should obtain it and follow its directions. Rescue breathing is not recommended. The technique for chest compressions is ideally taught with emphasis on a metronomeguided rate of 100 per minute. Additionally, full chest recoil after each compression is specifically emphasized.^8^

## Guidance from the three phases of cardiac arrest

Adoption of the cardio cerebral resuscitation technique will prompt some changes in EMS protocols; these are best understood in the context of the three phases of cardiac arrest due to VF. The three-phase time-dependent conception of cardiac arrest due to VF was articulated by Weisfeldt and Becker.^9^–^11^

**The electrical phase** is the first phase, lasting about 5 minutes. The most important intervention during this phase is defibrillation. This is why the availability of AEDs and programs to encourage their use have saved lives in a wide variety of settings, including airplanes airports, casinos, and the community^12^.

**The circulatory phase** is next. It varies in duration but runs approximately from minute 5 to minute 15 of VF arrest. During this time, generation of adequate cerebral and coronary perfusion pressure before and after defibrillation is critical to neurologically normal survival. Ironically, if anAED is the first intervention applied during this phase, the subject is much less likely to survive. If preshock chest compressions are not provided, defibrillation during the circulatory phase almost always results in a pulseless rhythm, asystole, or pulseless electrical activity. The previous stacked-shock protocol for the use ofAEDs resulted in prolonged interruption of essential chest compressions, not only for rhythm analysis before shocks but also for rhythm analysis after shocks during this circulatory phase of cardiac arrest.

Successful resuscitation from these pulseless rhythms requires not only preshock chest compressions but also prompt, effective post shock resumption of chest compressions.

**The metabolic phase** occurs late (sometime after15 minutes) in cardiac arrest due to VF. This is when resuscitative efforts are least successful and is the phase for which new innovative concepts are needed.

## Changes in cardiac life-support protocols

One reason why survival of out-of-hospital cardiac arrest has been so poor is that paramedics, who almost always arrive after the electrical phase of cardiac arrest due to VF, spend only half their time doing chest compressions.^13^ Interruptions are frequent because EMS personnel have been following existing guidelines. One of the more unfortunate recommendations of the old guidelines is the emphasis on stacked defibrillation, which results in a lack of chest compressions during prolonged and repeated analysis by AEDs during the circulatory phase of cardiac arrest due to VF—delays that have proved to be lethal. Similarly endotracheal intubation by EMS rescuers causes delay and disruption of the chest compressions. It also causes adverse effects related to positive pressure ventilation and frequent hyperventilation. In contrast, cardio cerebral resuscitation discourages endotracheal intubation during the electrical and circulatory phases of cardiac arrest due to VF.^13^,^14^

Defibrillator pad electrodes are applied and the patient is given 200 chest compressions and then a single defibrillation shock that is immediately followed by 200 more chest compressions before the rhythm and pulse are analyzed. These additional 200 chest compressions applied after the shock but before rhythm and pulse analysis represent another important aspect of cardio cerebral resuscitation. Therefore, chest compressions were immediately initiated until an arterial pressure was established.^9^

## A new approach to oxygenation

It has been documented that positive pressure ventilation during VF arrest is detrimental, concluding that “there is an inversely proportional relationship between mean Intrathoracic pressure, coronary perfusion pressure, and survival from cardiac arrest. Adverse effects of positive pressure ventilation include an increase in intrathoracic pressure as well as the inability to develop a negative intrathoracic pressure during the release phase of chest compression. Positive pressure ventilation inhibits venous return to the thorax and right heart, resulting in decreased coronary and cerebral pressures. Additionally, hyperventilation and increased intrathoracic pressure have adverse effects on intracranial pressure and cerebral perfusion pressure. These effects are compounded by the fact that ventilation rates by physicians and paramedic rescuers are often much faster than the rate recommended by the guidelines, even after extensive retraining.

During cardiac arrest, faster ventilation rates increase the mean intrathoracic pressure and further impede forward blood flow.^1^ Accordingly, cardio cerebral resuscitation recommends opening the airway with an oropharyngeal device, placement of a nonrebreather mask, and administration of high-flow (about 10 L/min) oxygen.^15^

Uninterrupted perfusion of the heart and brain prior to defibrillation during prolonged cardiac arrest is essential to neurologically normal survival. It is our conviction that the widespread implementation of cardio cerebral resuscitation for cardiac arrest will dramatically improve survival. This may mandate a paradigm shift away from advanced cardiac life support and basic life support, which emphasize standardization of content and format rather than institution- or agency- specific protocols and training.^16^–^18^
